# Lovastatin Inhibits HIV-1-Induced MHC-I Downregulation by Targeting Nef–AP-1 Complex Formation: A New Strategy to Boost Immune Eradication of HIV-1 Infected Cells

**DOI:** 10.3389/fimmu.2019.02151

**Published:** 2019-09-10

**Authors:** Bingfeng Liu, Xu Zhang, Wanying Zhang, Liyang Wu, Shuliang Jing, Weiwei Liu, Baijin Xia, Fan Zou, Lijuan Lu, Xiancai Ma, Dalian He, Qifei Hu, Yiwen Zhang, Kai Deng, Weiping Cai, Xiaoping Tang, Tao Peng, Hui Zhang, Linghua Li

**Affiliations:** ^1^Key Laboratory of Tropical Disease Control of Ministry of Education, Guangdong Engineering Research Center for Antimicrobial Agent and Immunotechnology, Zhongshan School of Medicine, Institute of Human Virology, Sun Yat-sen University, Guangzhou, China; ^2^Department of Infectious Diseases, Guangzhou Eighth People's Hospital, Guangzhou Medical University, Guangzhou, China; ^3^Sino-French Hoffmann Institute, Guangzhou Medical University, Guangzhou, China; ^4^Department of Molecular Therapy, Qianyang Biomedical Research Institute, Guangzhou, China; ^5^Guangzhou Women and Children Hospital, Institute of Pediatrics, Guangzhou Medical University, Guangzhou, China

**Keywords:** HIV-1, Nef, lovastatin, MHC-I, CD4, SERINC5, AP-1, immune surveillance

## Abstract

Current combined antiretroviral therapy (cART) mainly targets 3 of the 15 HIV proteins leaving many potential viral vulnerabilities unexploited. To purge the HIV-1 latent reservoir, various strategies including “shock and kill” have been developed. A key question is how to restore impaired immune surveillance. HIV-1 protein Nef has long been known to mediate the downregulation of cell-surface MHC-I and assist HIV-1 to evade the immune system. Through high throughput screening of Food and Drug Administration (FDA) approved drugs, we identified lovastatin, a statin drug, to significantly antagonize Nef to downregulate MHC-I, CD4, and SERINC5, and inhibit the intrinsic infectivity of virions. In addition, lovastatin boosted autologous CTLs to eradicate the infected cells and effectively inhibit the subsequent viral rebound in CD4^+^ T-lymphocytes isolated from HIV-1-infected individuals receiving suppressive cART. Furthermore, we found that lovastatin inhibits Nef-induced MHC-I downregulation by directly binding with Nef and disrupting the Nef–AP-1 complex. These results demonstrate that lovastatin is a promising agent for counteracting Nef-mediated downregulation of MHC-I, CD4, and SERINC5. Lovastatin could potentially be used in the clinic to enhance anti-HIV-1 immune surveillance.

## Introduction

The HIV-1 infection induces an immune response involving the development of anti-HIV CD8^+^ cytotoxic T lymphocytes (CTLs) (1). Though a significant number of the circulating CTL population is directed against HIV-1–infected cells, the virus is able to evade immune surveillance and establish latent reservoirs in resting CD4^+^ T cells despite the continuous combined antiretroviral therapy (cART) (2). During disease progression without any medical interference, the HIV-1 viral load eventually increases, destroying most of the CD4^+^ lymphocytes and leaving HIV-1 infected individuals increasingly susceptible to opportunistic infections (3).

To eliminate the infected cells by the CTLs, the members of the class I major histocompatibility complex (MHC-I) are required to present viral antigens on the cellular surface (4). HIV-1 requires its pathogenic factor, Nef, to enhance virion infectivity and maintain the high-titer replication *in vivo* (4). Nef is able to down-regulate cell-surface molecules, notably MHC-I, CD4, CD28, CCR5, SERINC3, and SERINC5 (5–8). Nef interacts directly with the cytoplasmic tail of MHC-I, which promotes the assembly of Nef/MHC-I/ adaptor protein 1 (AP-1) complexes via N-terminal WxxVxxxM_13−20_, 4E_62−65_, and PxxPxR_72−77_ motifs on Nef, to divert MHC-I from the default pathway to the plasma membrane (9, 10). Additionally, Nef also sequesters MHC-I at the paranuclear *trans* Golgi network (TGN) (11, 12). These strategies decrease the expression of MHC-I on cellular surface, allowing Nef to assist the virus-producing cells in immune evasion (13). In contrast to its effect on MHC-I, Nef downregulates CD4 molecule through its WLE_57−59_ and dileucine (ExxxLL_160−165_) based endocytosis motifs interaction with AP-2/clathrin complexes (14–16). This complexes mediate endocytosis from the plasma membrane to endosome/lysosome systems and eliminate the interference of viral receptors during HIV-1 maturation or release (17–19). Recent studies have demonstrated that the host transmembrane proteins SERINC3 and SERINC5 are potent inhibitors of virion infectivity (7, 8). Nef promotes viral infectivity by redirecting SERINC3/5 to the endosomal compartment and excluding them from virions (8, 20). Nef also utilizes similar functional motifs to downregulate both SERINC5 and CD4. The mutations in the G_2_, CAW_55−57_, RR_105, 106_, LL_164, 165_, and ED_178, 179_ residues on Nef abrogate the SERINC5 antagonism (20, 21). In addition, Nef can affect various cellular functions in different ways, including by alteration of T-lymphocyte activation and maturation through the interaction of its PxxPxR_72−77_ domain with SH3 domains of Src family kinases (SFKs), and subversion of the apoptotic machinery by blocking the Fas and TNFR signal pathways with the Nef–PI-3–PAK complexes (22–25).

Owing to its complex biology, the lack of well-defined *ex vivo* assay system hampers the development of potent inhibitors that are effective against a broad range of Nef activities. Several Nef-interacting small compounds and peptides have been identified and shown to target an SH3 binding surface and inhibit its interaction with Hck (26–30). In particular, one study identified that hydroxypyrazole-based Nef inhibitors can restore MHC-I in HIV-1 infected patient cells, and trigger the CTL response to eliminate the infected CD4^+^ T cells (26).

Although many compounds showed efficacy and were able to counteract MHC-I or CD4 downregulation, the binding affinity was low and these compounds were highly cytotoxic (31–33). Furthermore, few of them show any benefit in terms of recovering anti-HIV-1 immunosurveillance following reactivation of latent reservoir.

The “shock and kill” strategy has been extensively studied, and efforts to strengthen the reactivation and eradication of HIV-1 latency are ongoing (34). Ultimately, there is an urgent need for more potent agents that inhibit the Nef-mediated MHC-I downregulation. To facilitate the identification of such therapeutic agents, we performed a high throughput screen of clinically approved drugs and identified lovastatin as an efficacious HIV-1 Nef specific inhibitor with low cytotoxicity. Lovastatin has the potential to restore the MHC-I, CD4, and SERINC5 expression on cell surface. This compound can both inhibit the intrinsic infectivity of virions, which is enhanced by Nef, and boost CTL responses to eliminate HIV-1 infected cells. We also demonstrate that lovastatin exerts these functions by directly targeting Nef core region and physically blocking the formation of the Nef–AP-1 complexes.

## Materials and Methods

### Patient Cohort

This research was approved by the Ethics Review Board of The Eighth People's Hospital at Guangzhou (Guangzhou Infectious Disease Hospital, Guangzhou, China) and the Ethics Review Board of Sun Yat-Sen University. HIV-1–infected patients were recruited at The Eighth People's Hospital at Guangzhou and given written informed consent with approval of the Ethics Committees. All patients were recruited based on prolonged continuous suppression of plasma HIV-1 viremia on cART to below the limit of detection of standard clinical assays (<50 copies HIV-1 RNA ml^−1^). Unidentified human peripheral blood mononuclear cells (PBMCs) of healthy blood donors provided by the Guangzhou Blood Center. We did not have any interaction with these human subjects or protected information, and therefore no informed consent was required.

### Plasmids and Small Molecules

HIV-1 infectious clone pNL4-3 and its derivative pNL4-3-ΔEnv-EGFP were obtained through the NIH AIDS Reagent Program, Division of AIDS, NIAID, NIH. HIV-1 *nef* gene was amplified from pNL4-3 and the haemagglutinin (HA)-tag was inserted after the myristoylation motif (MGGKWS) at the 5′ terminus by PCR, then, the 5′-HA-Nef was ligated to pcDNA3.1 expressing vector. The site mutations on 5′ HA-*nef* were introduced by overlapping PCR as previously described (35). The green fluorescent protein (*gfp*) coding sequence (CDS) was tagged with HA tag at the 3′ terminus and constructed into the pcDNA3.1 vector. The *IRES-gfp* sequence was inserted into the 5′ HA-*nef* vector and named Nef-IRES-GFP. The *serinc5* was amplified from total cDNA of human PBMCs and the sequence was confirmed by sequencing. The *serinc5-gfp* fusion gene was generated by connection of two fragments with overlapping PCR and subcloned into pcDNA3.1 vector. pNL4-3-ΔNef and pNL4-3-ΔEnv/ΔNef-EGFP was constructed through introducing frame-shift mutation into the *nef* region of pNL4-3 and pNL4-3-ΔEnv-EGFP, respectively. All constructs were verified by DNA sequencing.

Lovastatin (PHR1285, Sigma) was used as indicated and its toxicity was determined by CCK-8 kit (Dojindo) according to manufacturer's instructions. Ethionamide (S1777), simvastatin (S1792), fluvastatin sodium (S1909), zoledronic acid (S1314), and pamidronate disodium (S1311) were purchased from Selluck Chemical and used as indicated. Lovastatin hydroxy acid (L472250) was purchased from J&K Chemicals.

### Cell Lines

HEK293T, HeLa, and TZM-bl cells were maintained in the conditioned Dulbecco's Modified Eagle medium (DMEM) medium (Gibco, Invitrogen, Carlsbad, CA) containing 10% fetal bovine serum (FBS) (Gibco, Invitrogen, Carlsbad, CA). All cell culture media contained 100 U ml^−1^ penicillin and 100 μg ml^−1^ streptomycin (Gibco, Invitrogen, Carlsbad, CA). All cell lines were maintained in an environment of 37°C and 5% CO_2_. Routine examination for mycoplasma verified that they all were mycoplasma-negative.

### Isolation and Culture of Primary Human T Lymphocytes

The PBMCs derived from healthy donors or HIV-1 infected patients were isolated from buffy-coat by Ficoll-Hypaque gradient separation. Excess PBMCs were cryopreserved until ready to use. Primary human CD4^+^ T cells were obtained from PBMCs by negative magnetic selection through Human CD4^+^ T Lymphocyte Enrichment Set-DM, BD-IMag^TM^. The isolated T cells were stimulated for 2 days with PHA-M at 1 μg ml^−1^ (Sigma) and recombinant human IL-2 at 10 ng ml^−1^ (R&D Systems) before HIV-1 infection. All cell culture media contained 100 U ml^−1^ penicillin and 100 μg ml^−1^ streptomycin (Gibco, Invitrogen, Carlsbad, CA) and cell culture were maintained in an environment of 37°C and 5% CO_2_.

### Enzyme-Linked Immunosorbent Assay (ELISA)

The HEK293T or CD4^+^ T cells (10^6^ cells) were treated with small compounds or vehicle at indicated concentrations in 24-well plates. Cells were collected and lysis after 48-h treatment. The quantity of human cholesterol in cells after drug-treatment was analyzed using QuickDetect^TM^ Total cholesterol (Human) ELISA Kit (catalog number K4431-100, BioVision) according to manufacturer's instructions. Viral particle production in cell cultures was determined with HIV-1 p24 ELISA kit by following the manufacturer's protocol (Clonetech).

### Flow Cytometry

For flow cytometry, the anti-HLA-A2-APC (BB7.2), anti-CD4-FITC (OKT4), anti-CD3-PE (UCHT1), and anti-CD8-Pacific blue (RPA-T8) were purchased from BD Biosciences, the anti-MHC-I-APC(W6/32) purchased from eBioscience. For intracellular HIV-1 Gag (p24) staining, surface staining was performed, then followed by intracellular staining for HIV-1 p24 (Santa Cruz Biotechnology) with the transcription factor buffer set according to the manufacturer's protocol (BD Biosciences). Data were acquired on a BD FACS aria and were analyzed with FlowJo software (Tree Star, Ashland, OR).

### Single Round Infectivity Assay

SERINC5 expressing or control HEK293T cells were seeded at 8 × 10^6^ cells per 100 mm dish. Twenty-four hours later, the pseudoviruses were generated by co-transfecting HEK293T cells with an envelope-expressing plasmid pcDNA3.1-Env_NL4−3_, plus pNL4-3-ΔEnv/ΔNef-EGFP or pNL4-3-ΔEnv-EGFP using calcium phosphate transfection system by following the manufacturer's instructions. Six hours after transfection, cells were treated with 4 μM lovastatin or vehicle. Twenty-four hours after transfection, cells were exchanged the fresh medium. Forty-eight hours after transfection, culture supernatants were harvested and filtered through a 0.22-μm membrane to remove cell debris. The p24 concentration of virus stocks were determined by p24 antigen ELISA. Relative infectivity of HIV-1 pseudoviruses was determined by luciferase assay system in 96-well TZM-bl cell which was performed as described earlier (7, 8). The infections were carried out in triplicates and the virus concentration used in infection was equivalent to the p24 concentration of 10 ng ml^−1^.

### Viral Outgrowth and Infection

Co-culture assays were performed to recover and amplify replication-competent viruses as previously described (36). Briefly, 1 × 10^6^ resting CD4^+^ T cells from HIV-1-infected individuals were stimulated by 1 × 10^7^ irradiated allogeneic PBMC from uninfected donors and the 1 μg ml^−1^ PHA-M at day 1, and typically, three additions of 5 × 10^6^ activated CD4^+^ lymphoblasts from uninfected donors as target cells were added for HIV-1 outgrowth at day 2, day 7, and day 14, respectively. The cells were cultured in RPMI-1640 media + IL-2 (10 ng ml^−1^, recombinant human, R&D Systems) all the time. After 14 days co-culture, the recovered viruses were harvested and tested for HIV-1 p24 protein. CD4^+^ T cells from HIV-1-infected individuals receiving suppressive cART were stimulated by adding PHA-M (1 μg ml^−1^) and IL-2 (10 ng ml^−1^) for 3 days. The activated CD4^+^ T cells from each patient were infected with the viruses recovered from the resting CD4^+^ T cells of same individual. The virus input used in infection was 10 ng ml^−1^ p24-equivalent. All infections were performed by centrifugation of target cells with viruses at 1,200 g for 1.5 h, followed by continuous culture in the RPMI-1640 media with IL-2 (10 ng ml^−1^).

### Co-culture of Autologous CD4^+^ and CD8^+^ T Cells

Three days after *in vitro* autologous HIV-1 infection, CD4^+^ T cells from infected individuals receiving suppressive cART were pre-treated with vehicle or lovastatin for 48 h, and then the cells were washed and mixed with autologous CD8^+^ T cells pre-stimulated with 800 ng ml^−1^ Gag-epitope peptides including EW10, WF9, HA9, TL9, TV9, and GL9 at a 1:1 ratio in the conditioned media at 5 × 10^6^ cells per ml (37). Every 2 days the cultures were tested for HIV-1 p24 protein with the HIV-1 p24 Antigen Assay kit by following the manufacturer's instructions. Eight days after co-culture, cells were stained with anti-CD3 and anti-CD8 antibodies, then permeabilized and stained for intracellular p24 Gag (Santa Cruz Biotechnology). Cells were analyzed by flow cytometry. Supernatants from each well were tested for HIV-1 p24 protein at various time points by ELISA via HIV-1 p24 antigen ELISA Kit (Clonetech), respectively.

### Lactate Dehydrogenase Assay

The specific killing activity of pre-stimulated CD8^+^ T cells toward autologous viral-infected CD4^+^ T cells at a 1:1 ratio was measured after co-culture for 8 days by lactate dehydrogenase assay using the CytoTox 96 non-radioactive cytotoxicity kit (G1781, Promega). The manufacturer's instructions were followed. Absorbance values of wells containing effector cells alone and target cells alone were combined and subtracted as background from the values of the co-cultures. Wells containing target cells alone were mixed with a lysis reagent for 30 min at 37°C and the resulting luminescence was set as 100% lysis. Cytotoxicity was calculated by using the following formula: % Cytotoxicity = (Experimental–Effector spontaneous–Target spontaneous)/(Target maximum–Target spontaneous) × 100%.

### Molecular Docking

Lovastatin or lovastatin hydroxy-acid docked with the crystal structure of HIV-1 Nef in complex with of AP-1 μ1 subunit (PDB:4emz) or AP-2 α/σ2 hemicomplex (PDB:4nee). First, the conformation of lovastatin or lovastatin hydroxy-acid was built by Chem3D Ultra. Second, the crystal structure of protein complex was prepared by removing unrelated water molecules. Then the whole protein complex was selected as docking pocket to calculate the compound docking pose by AutoDock 4.1/Autodock Vina in LigandScout 4.1.

### Co-immunoprecipitation

Co-immunoprecipitation was performed as described previously with minor modifications (38). Briefly, HEK293T cells were transfected with pcDNA3.1-Nef-HA, pcDNA3.1-Nef ^E63A^-HA, pcDNA3.1-Nef ^F68A^-HA, pcDNA3.1-Nef ^E63A/F68A^-HA, or pcDNA3.1-GFP-HA. After 48 h, the cells were disrupted in lysing buffer (150 mM NaCl, 10 mM Tris-HCl, 0.5% NP-40, 1% Triton X-100, 1 mM NaF, 1 mM Na_5_VO_4_, 2 mM EDTA, and 1% glycerol supplemented with PMSF and protease inhibitors) for 30 min on ice. Each lysate was immunoprecipitated with 40 μl of anti-HA agarose (E6779, Sigma) for 4 h. The beads were washed four times with STN buffer [10 mM Tris–HCl (pH 7.5), 0.25% NP-40 and 50 mM NaCl], and then boiled in SDS-containing buffer at 100°C for 10 min. The supernatants were then used for immunoblotting. Anti-HA (M180-3, MBL), Anti-AP1 (ab194384, Abcam), or anti-GAPDH (10494-1-AP, Proteintech) antibodies were used to detect their targets.

### Protein Purification

The plasmid pET32a harboring His-tagged Nef or Nef ^E63A/F68A^ genes were transformed into *E. coli* BL21(DE3) competent cells (Novagen), respectively. After the expression of proteins was induced by 1 mM isopropylthio-b-D-galactoside, the bacterial cells were lysed by sonication. The insoluble fraction was pelleted at 12,000 × g for 15 min, and the supernatant was applied to a Ni-conjugated agarose bead column (GE). After washing, the bound His fusion proteins were eluted with 500 mM imidazole. Then the proteins were suspended in PBS buffer and the concentration was measured by the Bradford method. The samples were then aliquoted and frozen at −80°C.

### Surface Plasmon Resonance

The measurements were carried out with a Biacore T100 instrument (GE Healthcare). A Biacore CM5 Sensor Chip and an amine coupling kit were purchased directly from GE Healthcare. The suitable pH value of 4.5 for His-Nef or His-Nef ^E63A/F68A^ immobilization (20 μg/ml in 10 mM acetate buffer) was determined first. The CM5 censor chip was activated and then injected with His-Nef and His-Nef ^E63A/F68A^ (20 μg/ml, in 10 mM acetate buffer, pH 4.5) for 7 min into channel 2 and 4, respectively. The residual activated groups on the surfaces were blocked with an injection of ethanolamine HCl (1 M) for 7 min. The lovastatin or fluvastatin was diluted at the indicated range of concentrations. Binding to the His-Nef or His-Nef ^E63A/F68A^ protein was monitored for about 60 s. The dissociate time was 120 s for lovastatin or fluvastatin with running buffer in per cycle.

## Results

### High Throughput Screen of Nef Inhibitors That Restore MHC-I Expression

**Figure 1:**
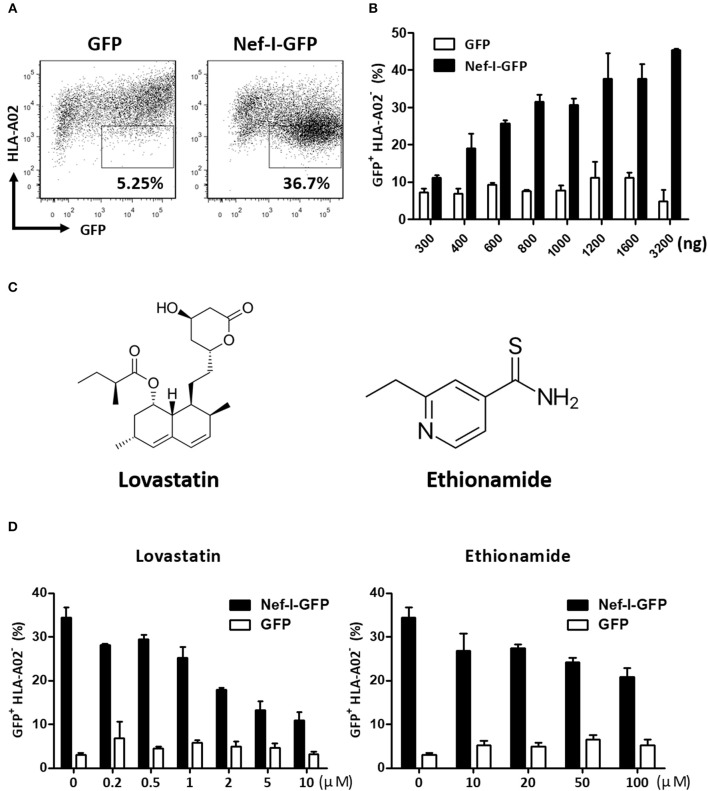
Screen and identification of small molecular compounds inhibiting HIV-1 Nef-mediated MHC-I down-regulation. HEK293T cells were transfected with pcDNA3.1-Nef-IRES-GFP or pcDNA3.1-IRES-GFP (800 ng per well), At 48 h post-transfection, Nef-mediated MHC-I down-regulation was analyzed by flow cytometry for staining of MHC-I **(A)**. The ratios of GFP ^+^ MHC-I^−^ HEK293T cells after transfection of pcDNA3.1-Nef-IRES-GFP or pcDNA3.1-IRES-GFP at different concentrations were summarized with bar plot. Data show the means ± standard deviations in three independent experiments **(B)**. Structures of lovastatin and ethionamide **(C)**. Twelve hours after transfection of Nef-I-GFP or empty control plasmid, HEK293T cells were treated with lovastatin or ethionamide at different concentrations. Forty-eight hours after transfection, FACS analysis was performed for the percentages of GFP^+^ MHC-I^−^ cells. Data show the means ± standard deviations in three independent experiments **(D)**.

A flow cytometry cell-based high-throughput screen was performed. Briefly, the Nef-expressing plasmid (Nef-IRES-GFP) or control plasmid (GFP) were transfected into HEK293T cells. In this assay, the expression of Nef caused downregulation of cell surface MHC-I (Figures 1A,B). The levels of GFP^+^ MHC-I^−^ in the cells reflected the Nef-mediated MHC-I downregulation and was measured by flow cytometry (Figure 1B). This assay model was adapted to identify inhibitors for Nef-mediated MHC-I downregulation by treatment with a United States Food and Drug Administration (FDA) approved drug library composed of 1,600 compounds at 50 μM. After 48 h, the wells containing GFP-positive cells were stained with an anti-MHC-I antibody and detected by flow cytometry. After the positive hits were confirmed by the same system, 32 hit compounds were found to markedly counteract the Nef-mediated down-regulation of MHC-I molecule. We then tested these 32 hit compounds to determine dose-dependency and selected 2 compounds, lovastatin and ethionamide, which showed >50% inhibition at 50 μM (Figure 1C). Both compounds displayed specific and potent effect against HIV-1 Nef and restored MHC-I density on the cell surface. Notably, flow cytometry data indicated that lovastatin exhibited superior Nef-inhibitory efficacy at lower concentrations than ethionamide (Figure 1D). Therefore, we chose to use lovastatin for subsequent experiments.

### Lovastatin Potently Inhibits Nef-Mediated Downregulation of MHC-I

**Figure 2:**
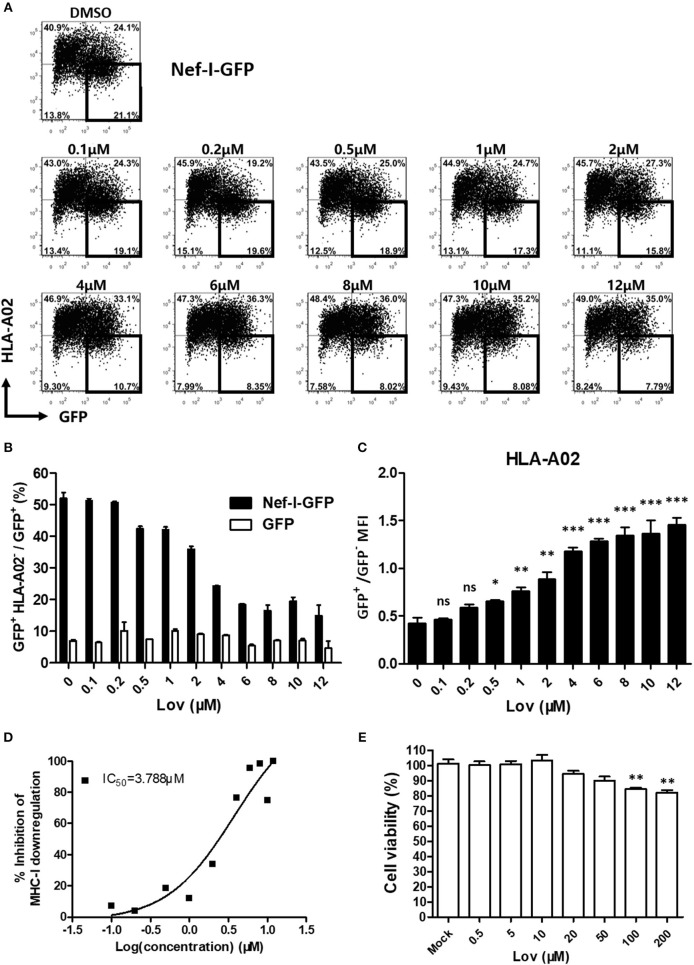
Lovastatin potently represses the ability of Nef to downregulate MHC-I. Twelve hours after transfection of pcDNA3.1-Nef-IRES-GFP (800 ng per well), HEK293T cells were treated with lovastatin from 0.1 to 12 μM. Forty-eight hours after transfection, FACS analysis was performed for the percentages of GFP^+^ MHC-I^−^ cells **(A)**. The ratios of GFP ^+^ MHC-I^−^ to GFP ^+^ population from pcDNA3.1-Nef-IRES-GFP or pcDNA3.1-IRES-GFP transfections were summarized with bar plot. Data show the means ± standard deviations in three independent experiments **(B)**. The ratios of MHC-I MFI on GFP^+^ to GFP^−^ cells from pcDNA3.1-Nef-IRES-GFP transfections were summarized with bar plot. Data show the means ± standard deviations in three independent experiments **(C)**. The IC50 was calculated according to above data of flow cytometry using GraphPad Prism software **(D)**. Human PBMCs were treated with lovastatin with the given concentrations for 48 h, and the cell viability was then measured by CCK-8 kit. Data show the means ± standard deviations in three independent experiments **(E)**. *P*-values were calculated using the two tailed unpaired Student's *t*-test with equal variances, *n* = 3. *p < 0.05, **p < 0.01, ***p < 0.001.

Lovastatin is a FDA approved statin drug used for lowering cholesterol in patients with hypercholesterolemia in order to reduce the risk of cardiovascular disease (39). Lovastatin effectively inhibits Nef-mediated downregulation of MHC-I in a dose-dependent manner (Figures 2A–C, Figure S1), with an IC_50_ of 3.788 μM (Figure 2D). Meanwhile, drug-treatment did not affect the Nef expression (Figure S2). To determine any cytotoxic effects, the viabilities of primary PBMCs and HEK293T cells were assessed by treatment with increasing concentrations of lovastatin. The results indicated that lovastatin at the concentration range inhibiting Nef-dependent MHC-I downregulation exhibited no measurable cell toxicity. A quite limited effect on cell viability could be found at higher concentrations up to 100 μM (Figure 2E, Figure S3).

### Lovastatin Inhibits Nef-Mediated Downregulation of CD4 and SERINC5, and the Intrinsic Infectivity of Virions

**Figure 3:**
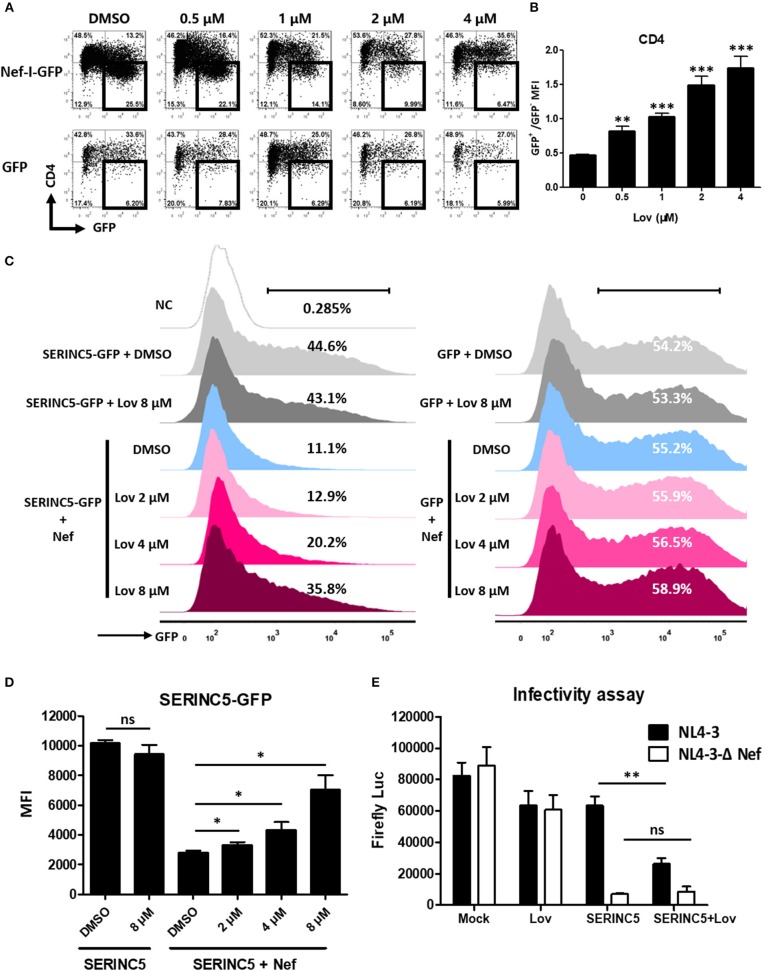
Lovastatin inhibits the ability of Nef to down-regulate CD4 and SERINC5, and the intrinsic infectivity of virions. TZM-bl cells were transfected with pcDNA3.1-Nef-IRES-GFP or pcDNA3.1-IRES-GFP (800 ng per well). Twelve hours after transfection, the cultures were treated with lovastatin with the given concentrations. Forty-eight hours after transfection, the percentages of the GFP^+^ CD4^−^ cells were analyzed by flow cytometry **(A)**. The ratios of CD4 MFI on GFP^+^ to GFP^−^ cells from pcDNA3.1-Nef-IRES-GFP transfections were summarized with bar plot. Data show the means ± standard deviations in three independent experiments **(B)**. Twelve hours after transfection of SERINC5-GFP/GFP (500 ng per well) and HA-Nef plasmids (500 ng per well), HEK293T cells were treated with lovastatin or vehicle at indicated concentrations, FACS analysis was performed for the percentages of the GFP ^+^ cells at 48 h after transfection **(C)**. The MFI of SERINC5-GFP fusion protein determined by flow cytometry. Data show the means ± standard deviations in three independent experiments **(D)**. Viruses were generated by co-transfection of HIV-1 Env-expressing plasmid and proviral HIV plasmids (pNL4-3-ΔEnv/ΔNef-EGFP or pNL4-3-ΔEnv-EGFP) into SERINC5-expressing or control HEK293T cells with treatment of lovastatin or vehicle. Forty-eight hours after transfection, culture supernatants were harvested. The infectivity of HIV-1 viruses was determined by luciferase assay system in 96-well TZM-bl cell with 10 ng p24-equivalent input viruses **(E)**. *P*-values were calculated using the two tailed unpaired Student's *t*-test with equal variances, *n* = 3. *p < 0.05, **p < 0.01, ***p < 0.001.

Previous reports have suggested that Nef also significantly decreases the expression of cell surface CD4 (5, 7). Accordingly, we treated Nef overexpressing TZM-bl cells with lovastatin or vehicle, and measured CD4 expression on the cell surface. We also found that lovastatin prevented Nef-induced CD4 downregulation (Figures 3A,B). As Nef utilizes a similar mechanism to downregulate SERINC5 and CD4 (20, 21), we thought it would be interesting to investigate whether lovastatin could also rescue SERINC5 expression. To this end, we constructed a SERINC5-GFP fusion protein whose expression was not affected by lovastatin (Figure 3C). We confirmed that the total amount of SERINC5-GFP fusion protein is also antagonized by Nef. Following this, SERINC5-downregulated cells were treated with lovastatin at the indicated concentrations. We found that lovastatin restored SERINC5-GFP expression by more than 3-fold at 8 μM (Figures 3C,D). Moreover, to further evaluate whether lovastatin could interfere with increased infectivity due to Nef, viruses were generated by co-transfection of HIV-1 Env-expressing plasmid and proviral HIV plasmids (pNL4-3-ΔEnv/ΔNef-EGFP or pNL4-3-ΔEnv-EGFP) into SERINC5-expressing HEK293T cells in the absence or presence of lovastatin. Then, the infectivity of the produced viruses was titrated using TZM-bl indicator cells. Ectopic expression of SERINC5 in viral-producing cells resulted in a 10-fold inhibition of HIV-1_NL4−3Δ*Env*/Δ*Nef*_ infectivity, while Nef maintained the HIV-1_NL4−3Δ*Env*_ viral infectivity (Figure 3E). However, lovastatin treatment produced a 2-fold decrease in the luciferase expression of the HIV-1_NL4−3Δ*Env*_, whereas the luciferase expression of HIV-1_NL4−3Δ*Env*/Δ*Nef*_ was only slightly affected (Figure 3E). These results indicate that lovastatin treatment prevents Nef-mediated downregulation of SERINC5 and subsequently influences the intrinsic infectivity of the viruses (Figure 3E).

### Lovastatin Counteracts Downregulation of MHC-I and CD4 Molecules During Wild-Type HIV-1 Infection

**Figure 4:**
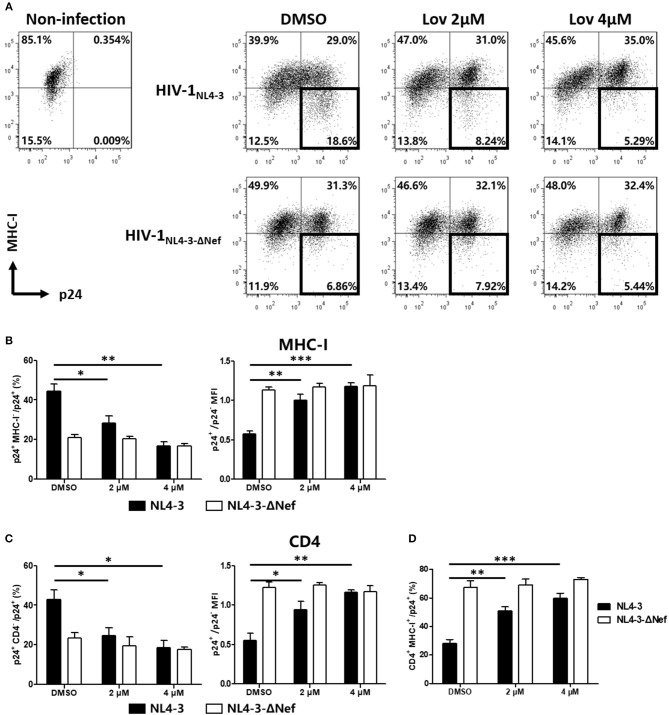
Lovastatin restores the MHC-I and CD4 on cell surface after the HIV-1_NL4−3_ infection. The activated primary CD4^+^ T cells were infected with HIV-1_NL4−3_ or HIV-1_NL4−3Δ*Nef*_ (p24 titer of 100 ng ml^−1^). At day 3 post-infection, the cultures were treated with vehicle or lovastatin at 37°C for 48 h. HIV p24 and MHC-I expressions gated on CD3^+^ CD8^−^ subpopulation were analyzed by flow cytometry **(A)**. The percentages of p24^+^ MHC-I^−^ subpopulation and the ratios of MHC-I MFI on GFP^+^ to GFP^−^ cells from the above flow cytometry analysis were summarized with bar plots **(B)**. The percentages of p24^+^ CD4^−^ subpopulation, the ratios of CD4 MFI on GFP^+^ to GFP^−^ cells **(C)**, and the percentages MHC-I^+^ CD4^+^ subpopulation **(D)** were summarized with bar plots. Data show the means ± standard deviations in three independent experiments. *P*-values were calculated using the two tailed unpaired Student's *t*-test with equal variances, *n* = 3. *p < 0.05, **p < 0.01, ***p < 0.001.

Next, we asked whether lovastatin could interfere with the downregulation of cell surface molecules induced by wild-type HIV-1 in primary CD4^+^ T-cells. We infected primary CD4^+^ T lymphocytes with wild-type HIV-1_NL4−3_ or Nef-deficient HIV-1_NL4−3−Δ*Nef*_. At day three post-infection, the cultures were treated with vehicle or lovastatin. Forty-eight hours later, the cells were analyzed for cell-surface MHC-I and CD4 by flow cytometry (Figure 4, Figure S4). In wild-type HIV-1–infected T-lymphocyte populations (CD3^+^ CD8^−^), the ratio of p24^+^ MHC-I^−^/p24^+^ cells decreased from 44 to 16%, and CD4 negative cells decreased from 42 to 19% with 4 μM lovastatin treatment, meanwhile MHC-I and CD4 double positive lymphocytes increased approximately 2-fold (Figures 4B–D). The mean fluorescence intensities (MFI) of MHC-I and CD4 of p24^+^ cells also significant increased after lovastatin treatment (Figures 4B,C). Moreover, lovastatin did not significantly affect MHC-I or CD4 expression in Nef-deficient viral infection (Figure 4, Figure S4). We therefore concluded that, lovastatin significantly and selectively blocked MHC-I and CD4 downregulation on primary cells induced by HIV-1 Nef in a dose-dependent manner.

### Pre-treatment of Lovastatin Boosts Autologous CTL Response Against the Reactivated Latent Reservoir From HIV-1 Infected Individuals

**Figure 5:**
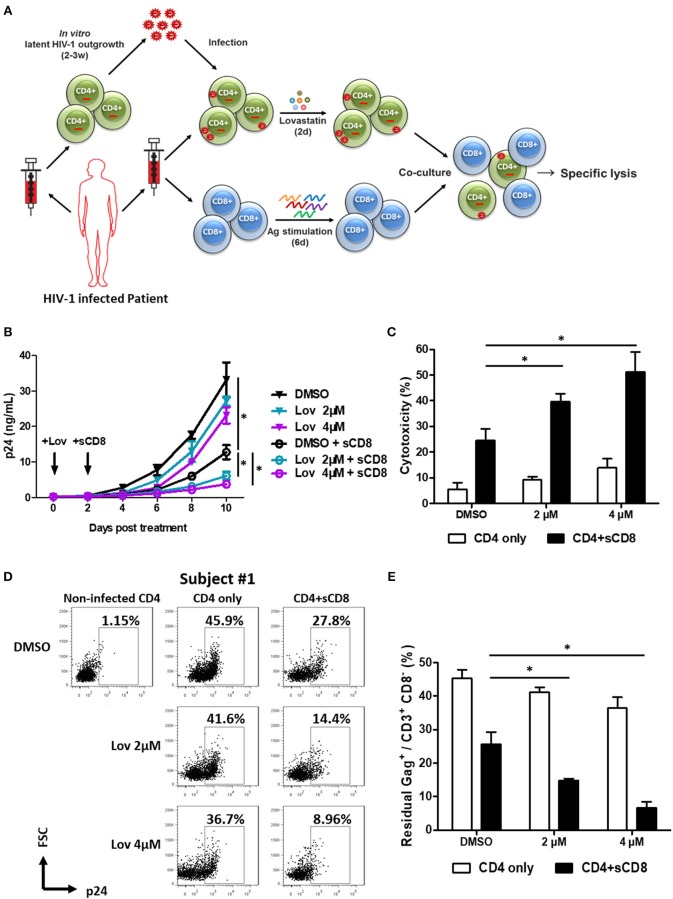
Pre-treatment of lovastatin boosts autologous CTL response against the reactivated latent reservoir from HIV-1 infected individuals. Experimental design. The activated CD4^+^ T cells from HIV-infected individuals receiving suppressive cART were infected with the viruses recovered from the resting CD4^+^ T cells of same patients, CD4^+^ T cells were incubated with lovastatin or vehicle for 48 h, then the cells were washed and mixed with autologous Gag peptides-stimulated CD8^+^ T cells at a 1:1 ratio **(A)**. Every 2 days the cultures were tested for HIV-1 p24 antigen by ELISA **(B)**. Eight days after co-culture, specific killing of infected CD4^+^ T cells by autologous CTLs was determined by LDH assay **(C)**. The residual Gag^+^ T cells gated on CD3^+^ CD8^−^ subpopulation were analyzed by flow cytometry **(D)**. The ratios of residual Gag^+^ to CD3^+^ CD8^−^ subpopulation were summarized from the above flow cytometry analysis **(E)**. Data show the means ± standard deviations from results of three HIV-1 infected individuals. *P*-values were calculated using the two tailed paired Student's *t*-test with equal variances, *n* = 3. *p < 0.05.

Since downregulation of cell-surface MHC-I molecules to paranuclear TGN/endosomal compartments allows HIV-1 to evade the immune system (40–42), we next asked whether lovastatin could reinforce host CTL response against HIV-1 infected cells. Therefore, we first isolated CD4^+^ T cells from HIV-1–infected individuals receiving suppressive cART and stimulated the outgrowth of replication-competent viruses from these cells. Then, activated CD4^+^ T cells were infected with the outgrown viruses recovered from the same patient. Three days after *in vitro* autologous HIV-1 infection, CD4^+^ T cells were pretreated with lovastatin for 48 h. These cells were then co-cultured with autologous CD8^+^ T cells pre-stimulated with a mixture of Gag-antigen epitope peptides including EW10, WF9, HA9, TL9, TV9, and GL9, which have been described as unmutated epitopes in chronically HIV-1–infected patients, to assess HIV-1-specific CTL response and cytolytic activity (Figure 5A) (37). In the groups without CD8^+^ T cells co-culture, the result indicated that expression of the p24 antigen was slightly reduced following lovastatin treatment (Figure 5B). When co-cultures with Gag-antigen impulsed CD8^+^ T cells were maintained over a length of time, viral replication was persistently and effectively inhibited in lovastatin-pretreated groups compared to control groups (Figure 5B). In addition, the CTL-mediated cytotoxicity of infected CD4^+^ T cells was increased from 24.6 to 51.3% with lovastatin treatment by measuring LDH release (Figure 5C). To exclude the influence of non-infected CD4^+^ T cells in the system, the residual CD3^+^ CD8^−^ Gag^+^ T cells were analyzed by intracellular staining. Although, Gag^+^ T cells were also slightly diminished after lovastatin treatment without co-culture, the CD8^+^ T cells pre-stimulated with the Gag peptide mixture killed nearly 40% autologous infected CD4^+^ T cells (Figures 5D,E, Figure S5). Moreover, in the lovastatin pre-treated groups, the stimulated CD8^+^ T cells could more efficiently kill infected CD4^+^ T cells and achieved an elimination rate of more than 68% (Figures 5D,E, Figure S5). The cytotoxic effect was enhanced to more than 80% by increasing the concentrations of lovastatin (Figures 5D,E, Figure S5). Together, these results demonstrate that lovastatin significantly enhances host CTL response against the HIV-1–infected cells.

### Lovastatin Directly Targets Nef and Blocks the Interaction Between Nef and AP-1

**Figure 6:**
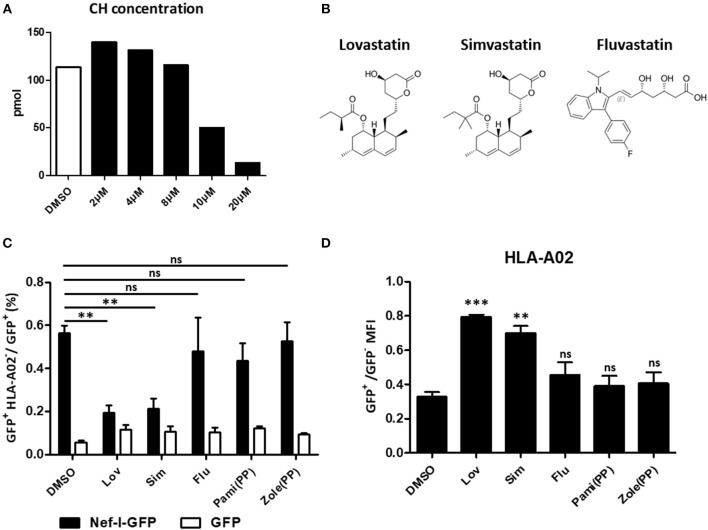
Restoration of MHC-I by lovastatin is independent from mevalonate pathway. HEK293T cells were treated with lovastatin from 2 to 20 μM. Forty-eight hours later, ELISA analysis the concentration of the cholesterol in the drug-treated cells **(A)**. Structures of lovastatin, simvastatin and fluvastatin **(B)**. Twelve hours after transfection of pcDNA3.1-Nef-IRES-GFP or pcDNA3.1-IRES-GFP (800 ng per well), HEK293T cells were treated with lovastatin, simvastatin, fluvastatin or bisphosphonates (zoledronic acid and pamidronate). Forty-eight hours after transfection, the ratios of GFP ^+^ MHC-I^−^ to GFP ^+^ population were analyzed by flow cytometry and summarized with bar plot. Data show the means ± standard deviations in three independent experiments **(C)**. The ratios of MHC-I MFI on GFP^+^ to GFP^−^ cells from pcDNA3.1-Nef-IRES-GFP transfections was determined by flow cytometry. Data show the means ± standard deviations in three independent experiments **(D)**. *P*-values were calculated using the two tailed unpaired Student's *t*-test with equal variances, *n* = 3. **p < 0.01, ***p < 0.001.

Lovastatin inhibits HMG-CoA reductase, the first committed enzyme of the mevalonate pathway which is involved in lowering cholesterol levels (Figure 6A). To understand whether the mevalonate pathway is involved in the mechanism of lovastatin induced MHC-I restoration, we chose three statin drugs and measured their effects toward the MHC-I downregulation induced by Nef, including lovastatin, simvastatin and fluvastatin (Figure 6B). The simvastatin, which has the same hexahydronaphthalen skeleton structure except the butyrate part, can also inhibit Nef-mediated downregulation of MHC-I at 4 μM, however, the fluvastatin, a hydroxy-acid and structurally different from lovastatin, failed to inhibit the Nef function to restore the MHC-I expression (Figures 6C,D, Figure S6). In addition, Nef-transfected HEK293T cells were treated with bisphosphonates including zoledronic acid or pamidronate, which also inhibit the mevalonate pathway and downstream isoprenoid biosynthesis (39). However, we found that the bisphosphonates have no significant impact on Nef-induced MHC-I downregulation (Figures 6C,D, Figure S6). Thus, restoration of MHC-I by lovastatin is independent from mevalonate pathway and the mechanism is related with its structure.

**Figure 7:**
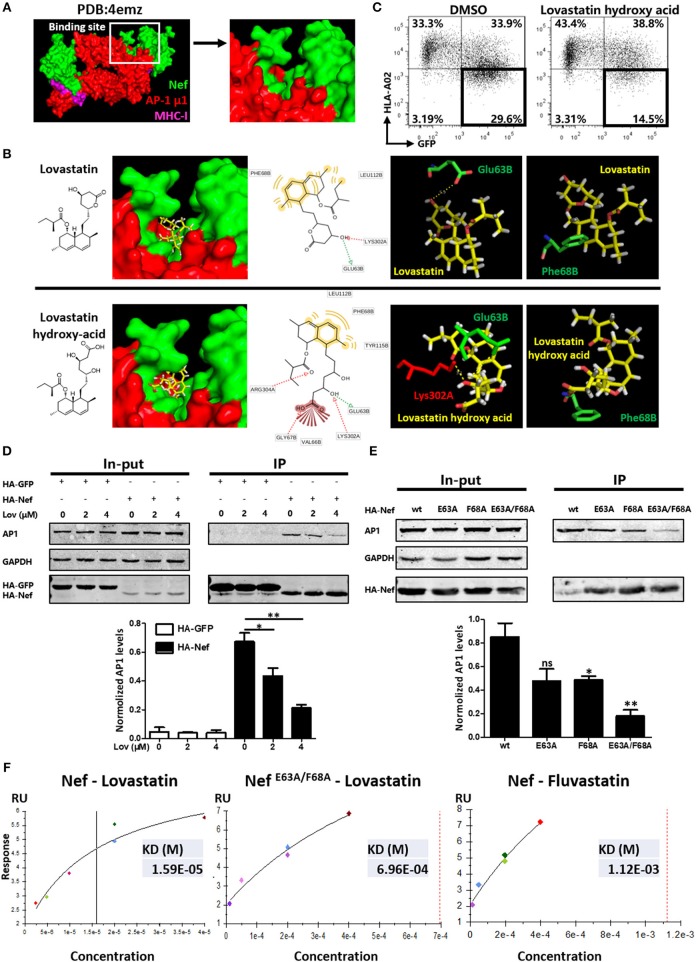
Lovastatin directly targets Nef and inhibits the interaction between Nef and AP-1. Molecular docking studies were performed to explore the binding modes of lovastatin or its hydroxy acid form with the crystal structure of Nef in complex with of AP-1 μ1 subunit and MHC-I cytoplasmic domain (PDB:4emz), the B and C chains in green belong to HIV Nef, the A and M chains in red belong to AP-1, and the D and E chains in purple belongs to MHC-I **(A)**. The pose of lovastatin or lovastatin hydroxy acid in binding pocket, the pharmacophore model, and the specific binding details of the interaction between ligand with Nef–AP-1 complex: the carbon backbone of lovastatin is in yellow, the Glu63 and Phe68 of chain B carbon backbone are in green and the Lys302 of chain A carbon backbones is in red **(B)**. Twelve hours after transfection of Nef-IRES-GFP (800 ng per well), HEK293T cells were treated with hydroxy acid of lovastatin at 4 μM. Forty-eight hours after transfection, MHC-I density on cell surface was analyzed by flow cytometry. These data represent three independent experiments **(C)**. HA-tagged Nef or GFP constructs were transfected into HEK293T cells. Twelve hours after transfection, cells were treated with lovastatin or vehicle. Forty-eight hours after transfection, cell lysates were immunoprecipitated with anti-HA antibody, and subsequently detected with anti-HA, anti-AP1 or anti-GAPDH antibody. HA-GFP as IP control, GAPDH was used as a loading control. Graph shows normalized levels of HA-Nef– or HA-GFP–interacted AP1 **(D)**. HA-tagged wild type Nef or mutant constructs were transfected into HEK293T cells. Forty-eight hours after transfection, cell lysates were immunoprecipitated with anti-HA antibody, and subsequently detected with anti-HA, anti-AP1 or anti-GAPDH antibody. GAPDH was used as a loading control. Graph shows normalized levels of HA-Nef–interacted AP1 **(E)**. Surface plasmon resonance experiments were performed to measure the binding affinity of lovastatin on Nef or Nef ^E63A/F68A^ and fluvastatin on Nef with a BIAcore T100 Biosensor System. These data represent two independent experiments **(F)**. *P*-values were calculated using the two tailed unpaired Student's *t*-test with equal variances, *n* = 3. *p < 0.05, **p < 0.01.

Previous studies have suggested that the formation of the Nef–MHC-I complex requires recruitment of the μ1 subunit of AP-1 (43). AP-1 is a cellular protein complex which is implicated in the TGN to endolysosomal pathways by linking clathrin to the cytoplasmic tails of cargo (44). Therefore, Nef-mediated AP-1 binding of MHC-I has been demonstrated to promote TGN accumulation and/or targeting of MHC-I into the endolysosomal pathway. To examine whether lovastatin affects this pathway, we performed the molecular docking studies of lovastatin with the crystal structure of HIV-1 Nef in complex with of AP-1 μ1 subunit (PDB:4emz), as well as the AP-2 α/σ2 hemicomplex (PDB:4nee) (Figures 7A,B, Figure S7). Then we selected the whole protein complex as a docking pocket to calculate the compound docking pose. We found that the top 5 poses for lovastatin were in the pocket located in the interface between Nef and AP-1. The naphthalene ring group of lovastatin has strong hydrophobic or aromatic interactions with Phe68 and Leu112 in Nef, the hydroxyl group of lovastatin lactone ring has hydrogen bond interaction with Glu63 in Nef and Lys302 in AP-1 as hydrogen-binding donor and receptor, respectively (Figure 7B). The binding pocket belongs to a globular core region (residues 58–149) of Nef. This region contains several critical residues and motifs for MHC-I and CD4 downregulation (45). As the lactone ring of lovastatin is hydrolyzed *in vivo* to the active, hydroxy-acid form. We also performed the molecular docking studies of lovastatin hydroxy-acid with Nef–AP-1 complex and found that the hydroxy-acid form was in the same binding pocket of Nef–AP-1 as lovastatin (Figure 7B). Moreover, hydroxy acid of lovastatin can also inhibit Nef-mediated downregulation of MHC-I (Figure 7C).

To further understand the mechanism by which lovastatin induces MHC-I restoration, we transfected Nef- or GFP-expressing plasmids into HEK293T cells and assayed for co-precipitation with AP-1. As shown in Figure 6, AP-1 readily co-precipitated with wild type Nef but not with GFP. However, Nef and AP-1 interaction was significantly inhibited by lovastatin in a dose-dependent manner (Figure 7D). Previous works indicated that the assembly of the Nef/MHC-I/AP-1 complex occurs via N-terminal WxxVxxxM_13−20_, 4E_62−65_ (the acidic domain), and PxxPxR_72−77_ motifs. Furthermore, the acidic domain has been shown to stabilize the interaction between Nef and AP-1 (9, 46–48). In combination with the docking data, we generated a mutated Nef in which Glu63 and Phe68 were replaced by an Ala (GCA) residue and each Nef mutant assayed for co-precipitation with AP-1. In contrast to wild-typed Nef, the Nef mutants (E63A and F68A), which did not influence the expression of Nef, attenuated the interaction between Nef and AP-1 while the E63A/F68A double mutant further decreased its binding to AP-1 (Figure 7E). In order to examine effects of these mutations toward MHC-I downregulation, wild-typed Nef and mutants were transfected to HEK293T cells. Flow cytometry data indicated that wild-typed Nef can mediate MHC-I down-modulation. However, E63A and F68A compromised this function, the ratio of GFP^+^ MHC-I^−^ population decreased from 34.9 to 25.7 or 23.2%, respectively. E63A/F68A double mutant further decreased this population (19.2%) (Figure S8). These results are in consistence with Figure 7E, since these mutations negatively impact this Nef–AP-1 interaction. In contrast, E63A or F68A on Nef did not significantly impact its function toward CD4 on cell surface, however, E63A/F68A double mutant partially attenuated the CD4 and SERINC5 down-regulation induced by Nef (Figure S9).

Surface plasmon resonance (SPR) was also performed using a BIAcore system to determine the binding affinity between Nef/Nef^E63A/F68A^ and lovastatin. The purified recombinant Nef-His or mutant protein (confirmed by SDS-PAGE) was immobilized onto a CM5 Sensor Chip (carboxymethylated dextran covalently attached to a gold surface) with an amine coupling kit. The SPR sensorgrams confirmed that Nef and lovastatin bind with a K_D_ of ~1.59 × 10^−5^ M, the binding affinity is much stronger than fluvastatin on Nef (K_D_ ~1.123 × 10^−3^ M). However, the K_D_ of lovastatin on Nef ^E63A/F68A^ mutant is about 6.96 × 10^−4^ M, suggesting that these mutations negatively impact this protein-ligand interaction compared with its wild-type (Figure 7F, Figure S10). Collectively, the Co-IP and SPR data confirm that lovastatin directly targets Nef and inhibits the Nef–AP-1 complex formation, and the Glu63 and Phe68 are required by Nef for its interaction with lovastatin and AP-1.

## Discussion

Although cART has been successful in prolonging the life span of HIV-infected individuals, it cannot purge the viral latent reservoir, which drives the search for novel anti-HIV agents to improve therapeutic effect, targeted either on the viral or host level. As immune evasion is an important strategy for HIV-1 survival *in vivo*, identification of compounds targeting these processes may promote efficient anti-HIV-1 immune surveillance (49). Some reports have indicated that atorvastatin effects the activation/exhaustion state in HIV-1–infected CD4^+^ T cells (50–52). In this study, we performed a high throughput screening of a clinically approved drug library and identified that lovastatin as a potent inhibitor of Nef-mediated downregulation of MHC-I and CD4. Nef-induced SERINC5 antagonism enhances the infectivity of the virion which is important for HIV-1 pathogenesis (8). There are currently no therapeutic agents that counteract this process. We also found that lovastatin inhibited the Nef-induced SERINC5 downregulation and the intrinsic infectivity of virions. Moreover, lovastatin boosts autologous CTL response against the reactivated HIV-1 latently-infected cells isolated from infected individuals. Herein, we have identified a potent small molecule inhibitor of Nef, which is already widely used in clinic.

The HIV-1 Nef structure contains a flexible N-terminal anchor domain (AN, residues 1–58), a core domain (Core, residues 58–149 and residues 180–206) and an internal flexible loop (FL, residues 149–179) (53–55). Nef can bind to the cytoplasmic tail of many membrane proteins, and alters their trafficking to the heterotetrameric clathrin AP-1, AP-2, and/or AP-3 (56, 57). AP-1 is thought to be important for trafficking between TGN and endosomes, and for eventual sorting of some proteins into lysosomes (58). Nef interacts with AP-1 and mediates MHC-I downregulation (9, 46–48). Lovastatin directly targets to Nef via interactions with Glu63 and Phe68 to induce inhibition of the Nef–AP-1 complexes formation and the putative binding pose of lovastatin are found in junction between Nef and AP-1.

Recent studies indicate that Nef uses similar functional motifs to downregulate SERINC5 and CD4 by redirecting them to the endosomal compartment and excluding them from virion particles (20, 21). In contrast, E63A or F68A on Nef did not significantly impact its function toward CD4 on cell surface, even E63A/F68A double mutant only partially restored the CD4 down-regulation induced by Nef, these data also suggest that additional residues may involve in the process (Figure S9). On the other hand, lovastatin is a pro-drug and its lactone ring is hydrolyzed *in vivo* to the active, hydroxy-acid form. According to our molecular docking studies, the naphthalene ring and hydroxyl group of this form shows strong interactions with multiple residues on Nef, including Glu63, Val66, Gly67, Phe68, Leu112, and Tyr115, which belong to the core region of Nef (Figure 7B). This region contains several critical residues and motifs for MHC-I and CD4 downregulation including (54).

Moreover, according to docking studies of lovastatin with Nef in complex with the AP-2 α/σ2 hemicomplex (PDB: 4NEE) (Figure S7). The naphthalene ring had hydrophobic interaction with the hydrophobic pocket composed by Leu112, Tyr115, and Phe121 in Nef, the hydroxyl group of lovastatin lactone ring had hydrogen bond interaction with Asp 108 in Nef and Ser297/Lys298 in AP-2. According to previous report, conserved HIV-1 Nef residues Asp108, Leu112, and Pro122 are identified as hot spots for inhibitor binding (27). The key residuals in our docking model are consistence with this report. Compared the pose of lovastatin with Nef–AP-1 and Nef–AP-2 complex in pharmacophore model, we found that lovastatin binding to these complexes via utilizing several common amino acids belong to a globular core region of Nef. Therefore, we suppose that the multiple loci interactions between the ligand and Nef core region may inhibit the interaction of Nef with AP-1, but also interrupt Nef to bind AP-2 complex. Therefore, lovastatin can antagonize Nef to downregulate MHC-I, as well as CD4/SERINC5.

Nef associates with the cell membrane mainly through myristoylation (59, 60). However, a previous study described Nef as a putative target for farnesyltransferase by the prenylation prediction suite (PrePS) (61). It is thought that prenylation might augment the membrane binding ability of Nef making it susceptible to suppression by HMG-CoA reductase inhibitors. However, prenylation enzymes recognize the CaaX box at the C-terminus of the target protein, most Nef sequences from various HIV-1 strains including HIV-1_NL4−3_ used in our study do not contain this motif. Therefore, the mechanism for the MHC-I restoration by lovastatin is unlikely to be through the inhibition of prenylation pathway. Furthermore, bisphosphonates, which also inhibit the mevalonate pathway and downstream isoprenoid biosynthesis, did not affect the downregulation function of Nef, thereby supporting this argument (39). It should also be noted that the association of Nef with lipid rafts in the Golgi networks could trap MHC-I and perhaps promote its association with required cellular proteins, such as AP-1 (62). Further studies are needed to determine whether lovastatin affect lipid rafts and Nef–MHC-I–AP-1 complex formation.

Although current anti-HIV drugs mainly target enzymes or structural proteins essential to viral replication and integration, HIV/SIV Nef is regarded as an accessory adaptor protein that fulfills many critical functions by interactions with multiple sequence motifs of host factors (4). The structural and functional insight gained in this study provide a potential candidate that might guide the efforts targeted at the generation of potent and clinically applicable Nef inhibitors. Future work is required for functional characterization and structure optimization on the Nef inhibitors provided in this study.

It has been reported that HIV-specific immunotherapy including CTLs could effectively target HIV-1-infected CD4^+^ T lymphocytes (63). The re-establishment of immune surveillance could be a feasible approach for achieving long-term suppression of the reactivation of viral latent reservoir without the continuation of cART (34, 64, 65). Accordingly, the enhancement of the specific immune recognition and cytotoxicity is required to thoroughly eradicate the viral latent reservoir *in vivo* (26, 66). One of the key questions is whether any special method could restore the downregulation of MHC-I induced by Nef. Our data found the lovastatin could not only affect infectivity, but also reinforce host CTL responses against the reactivated latent reservoir, suggesting that highly efficacious Nef inhibitors in combination with immune cell therapies should be evaluated in future HIV-1 clinical trials for viral reservoir eradication.

## Conclusion

HIV-1 protein Nef has several biological functions, including controlling the downregulation of cell-surface MHC-I and assisting HIV-1 in the evasion of the immune system. Here, we identified a small molecule inhibitor, lovastatin, which effectively inhibits Nef to downregulate MHC-I, CD4, and SERINC5. We also found that lovastatin inhibits the intrinsic infectivity of virions. Importantly, lovastatin can boost the activity of HIV-1-specific CTLs to eliminate reactivated latently-infected primary CD4^+^ T-lymphocytes and persistently suppress viral replication. Recent advances in anti-HIV-1 immunotherapy have brought us closer to the realizing possibility of eradicating HIV-1 reservoir. Our study proposes that the clinically approved drug, lovastatin, could potently lead us one step closer to this goal.

## Data Availability

The raw data supporting the conclusions of this manuscript will be made available by the authors, without undue reservation, to any qualified researcher.

## Ethics Statement

This study was carried out in accordance with the recommendations of the Ethics Review Board of The Eighth People's Hospital at Guangzhou (Guangzhou Infectious Disease Hospital) and the Ethics Review Board of Sun Yat-Sen University with written informed consent from all subjects. All subjects gave written informed consent in accordance with the Declaration of Helsinki. The protocol was approved by the Ethics Review Board of Guangzhou Eighth People's Hospital.

## Author Contributions

BL and XZ designed the experiments, performed most of these experiments, analyzed the data, and manuscript writing. WZ, SJ, WL, BX, FZ, LLu, XM, DH, and QH performed some of the experiments. LW carried out the bioinformatics and structural analysis. YZ, KD, WC, and XT provided scientific expertise and the interpretation of data for the work. LLi, HZ, and TP contributed to the idea generation, experimental design, manuscript writing and conceived the project.

### Conflict of Interest Statement

The authors declare that the research was conducted in the absence of any commercial or financial relationships that could be construed as a potential conflict of interest.
