# Supplementary material for: Lovastatin Inhibits HIV-1-Induced MHC-I Downregulation by Targeting Nef–AP-1 Complex Formation: A New Strategy to Boost Immune Eradication of HIV-1 Infected Cells
Source: Front Immunol. 2019 Sep 10;10:2151. doi: 10.3389/fimmu.2019.02151 (PMC6749138; doi:10.3389/fimmu.2019.02151)
Supplement: Supplementary file 1 [file Data_Sheet_1.docx]

**SUPPLEMENTARY FIGURES**

**Supplementary Figure 1**

**
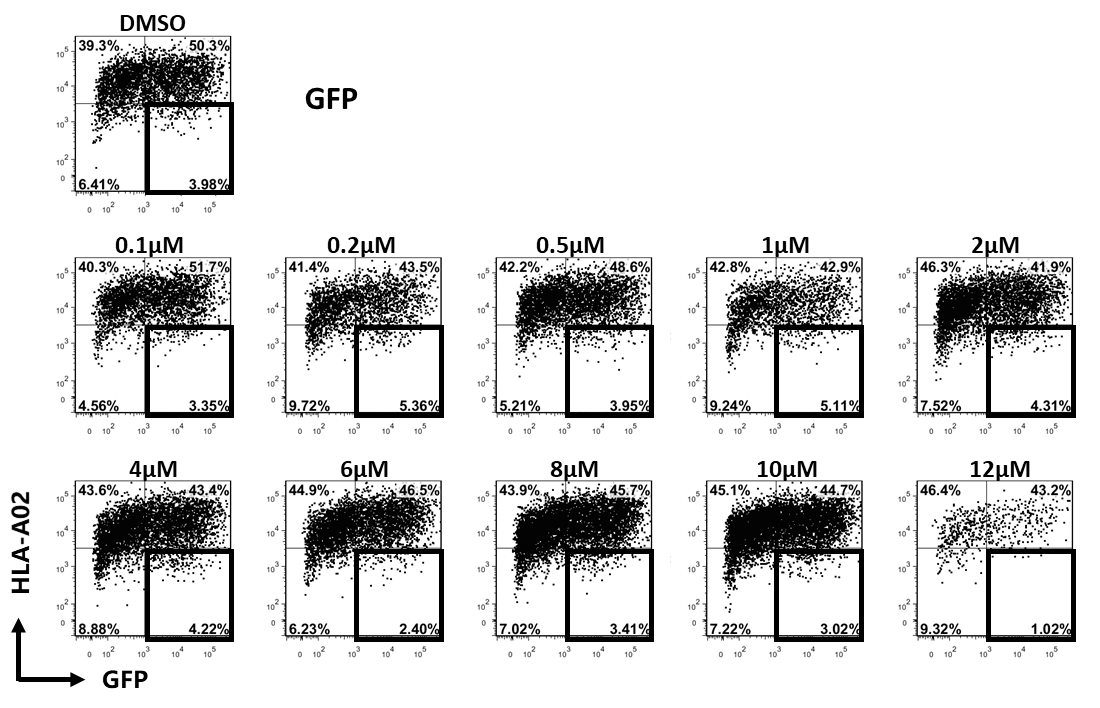
**

**Supplementary Figure 1. Lovastatin potently represses the ability of Nef to downregulate MHC-I.** Twelve hours after transfection of pcDNA3.1-IRES-GFP (800 ng per well), HEK293T cells were treated with lovastatin from 0.1 μM to 12 μM. Forty-eight hours after transfection, FACS analysis was performed for the percentages of GFP ^+^ MHC-I^-^ cells.

**Supplementary Figure 2**

**
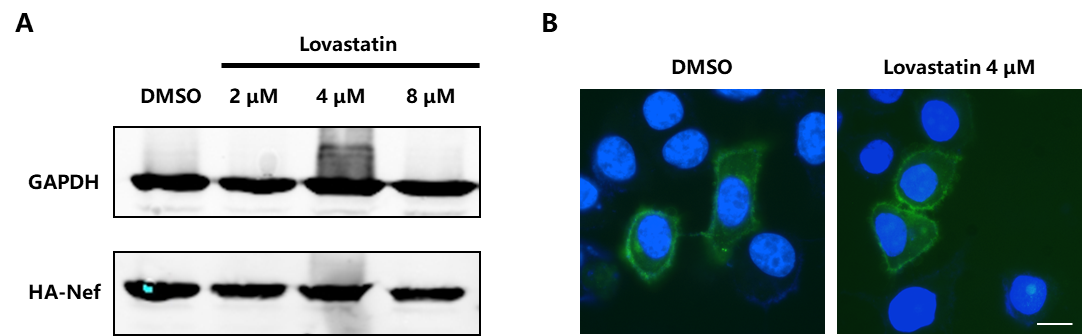
**

**Supplementary Figure 2. The lovastatin did not affect the Nef expression.** HEK293T cells were transfected with pcDNA3.1-Nef-HA (800 ng per well), twelve hours after transfection, cells were treated with lovastatin at different concentrations. Forty-eight hours after lovastatin treatment, the cells were collected, and western blotting was performed to detect the expression of Nef with anti-HA tag antibody in HEK293T cells **(A)**. Immunofluorescence staining was performed to detect the Nef-HA expression in HeLa cells with anti-HA tag antibody. DAPI staining represents the nuclei. Scale bars, 10 μm **(B)**.

**Supplementary Figure 3**

**
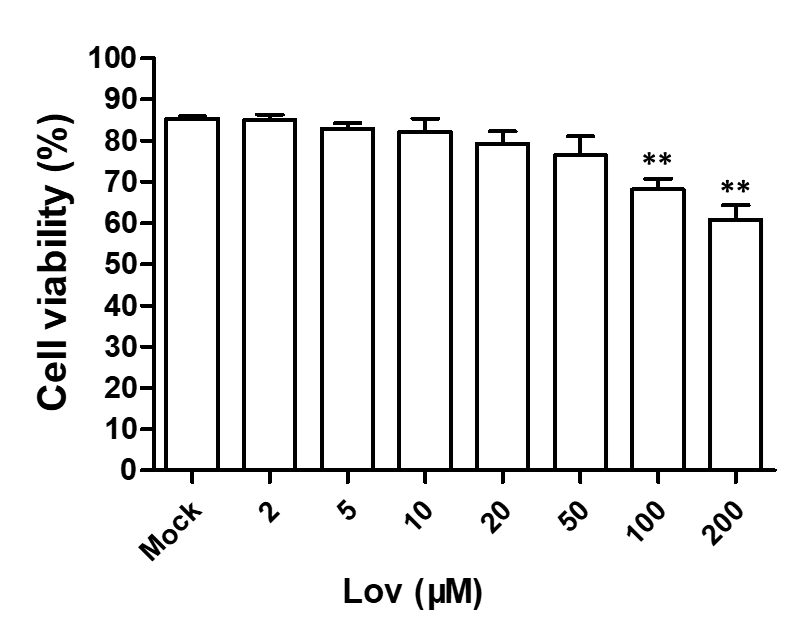
**

**Supplementary Figure 3. Evaluation of cytotoxicity on transfected HEK293T cells by lovastatin treatment.** HEK293T cells were transfected with pcDNA3.1-Nef-IRES-GFP (800 ng per well), Twelve hours after transfection, cells were treated with lovastatin at different concentrations. At 48 hours post-transfection, the cell viability was then measured by CCK-8 kit. Data show the means ± standard deviations in three independent experiments. P-values were calculated using the two tailed unpaired Student’s t-test with equal variances, n = 3. *p < 0.05, **p < 0.01, ***p < 0.001.

**Supplementary Figure 4**

**
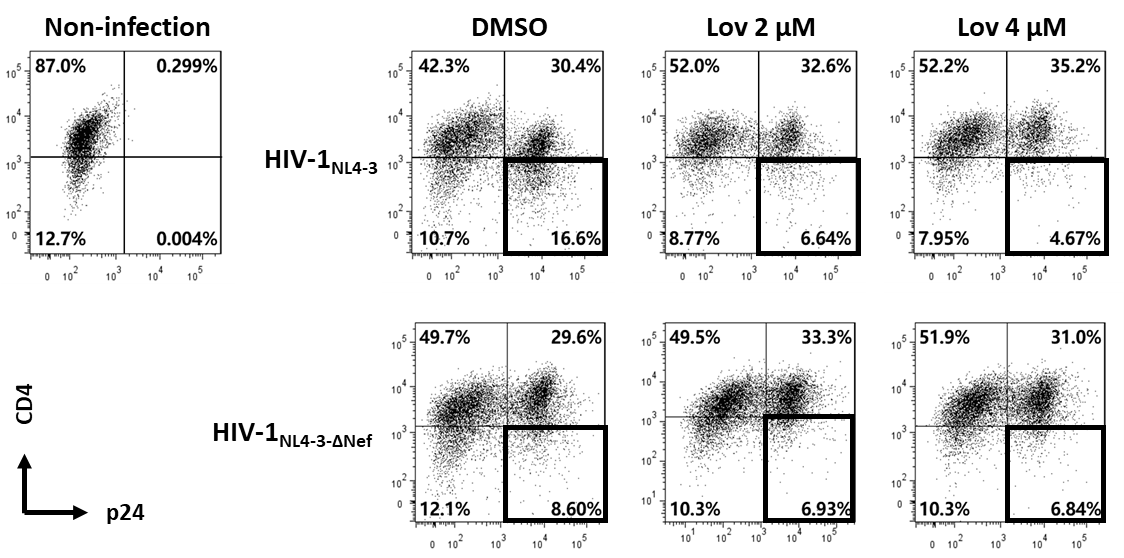
**

**Supplementary Figure 4.** **Lovastatin restores the CD4 on cell surface after the HIV-1_NL4-3_ infection.** The activated primary CD4^+^ T cells were infected with HIV-1_NL4-3_ or HIV-1_NL4-3ΔNef_ (p24 titer of 100 ng ml^-1^), At day 3 post-infection, the cultures were treated with vehicle or lovastatin at 37 °C for 48 hours. HIV p24 and CD4 expressions gated on CD3^+^ CD8^-^ subpopulation were analyzed by flow cytometry.

**Supplementary Figure 5**

**
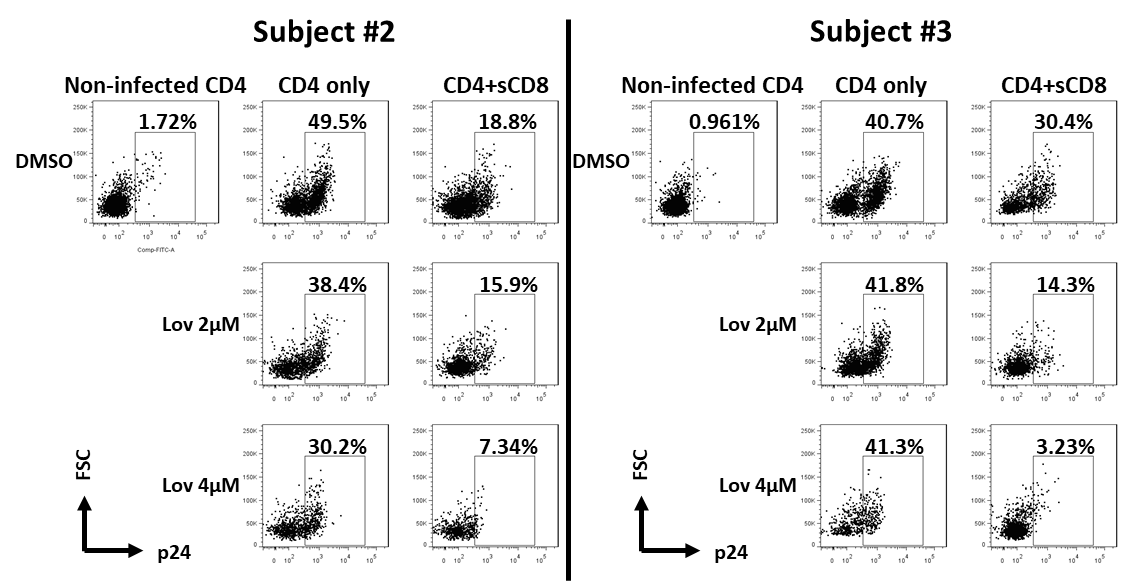
**

**Supplementary Figure 5. Pre-treatment of lovastatin boosts autologous CTL response against the reactivated latent reservoir from HIV-1 infected individuals.** The activated CD4^+^ T cells from HIV-infected individuals receiving suppressive cART were infected with the viruses recovered from the resting CD4^+^ T cells of same patients, CD4^+^ T cells were incubated with lovastatin or vehicle for 48 hours, then the cells were washed and mixed with autologous Gag peptides-stimulated CD8^+^ T cells at a 1:1 ratio. Eight days after co-culture, specific killing of infected CD4^+^ T cells by autologous CTLs was determined by the residual Gag^+^ T cells gated on CD3^+^ CD8^-^ subpopulation were analyzed by flow cytometry **(D)**.

**Supplementary Figure 6**

**
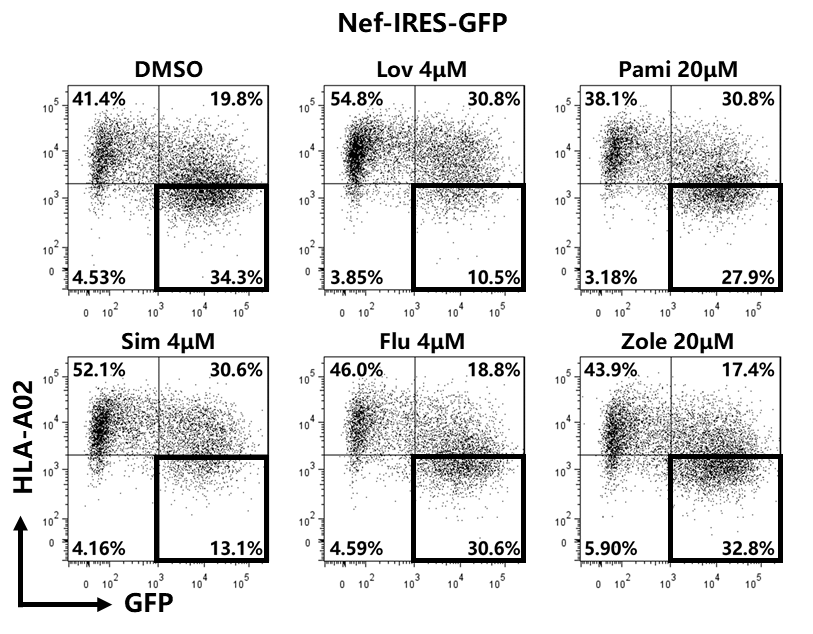
**

**Supplementary Figure 6. Restoration of MHC-I by lovastatin is independent from mevalonate pathway.** Twelve hours after transfection of pcDNA3.1-Nef-IRES-GFP (800 ng per well), HEK293T cells were treated with lovastatin, simvastatin, fluvastatin or bisphosphonates (zoledronic acid and pamidronate). Forty-eight hours after transfection, FACS analysis was performed for the percentages of GFP ^+^ MHC-I^-^ cells. These data represent three independent experiments.

**Supplementary Figure 7**

**
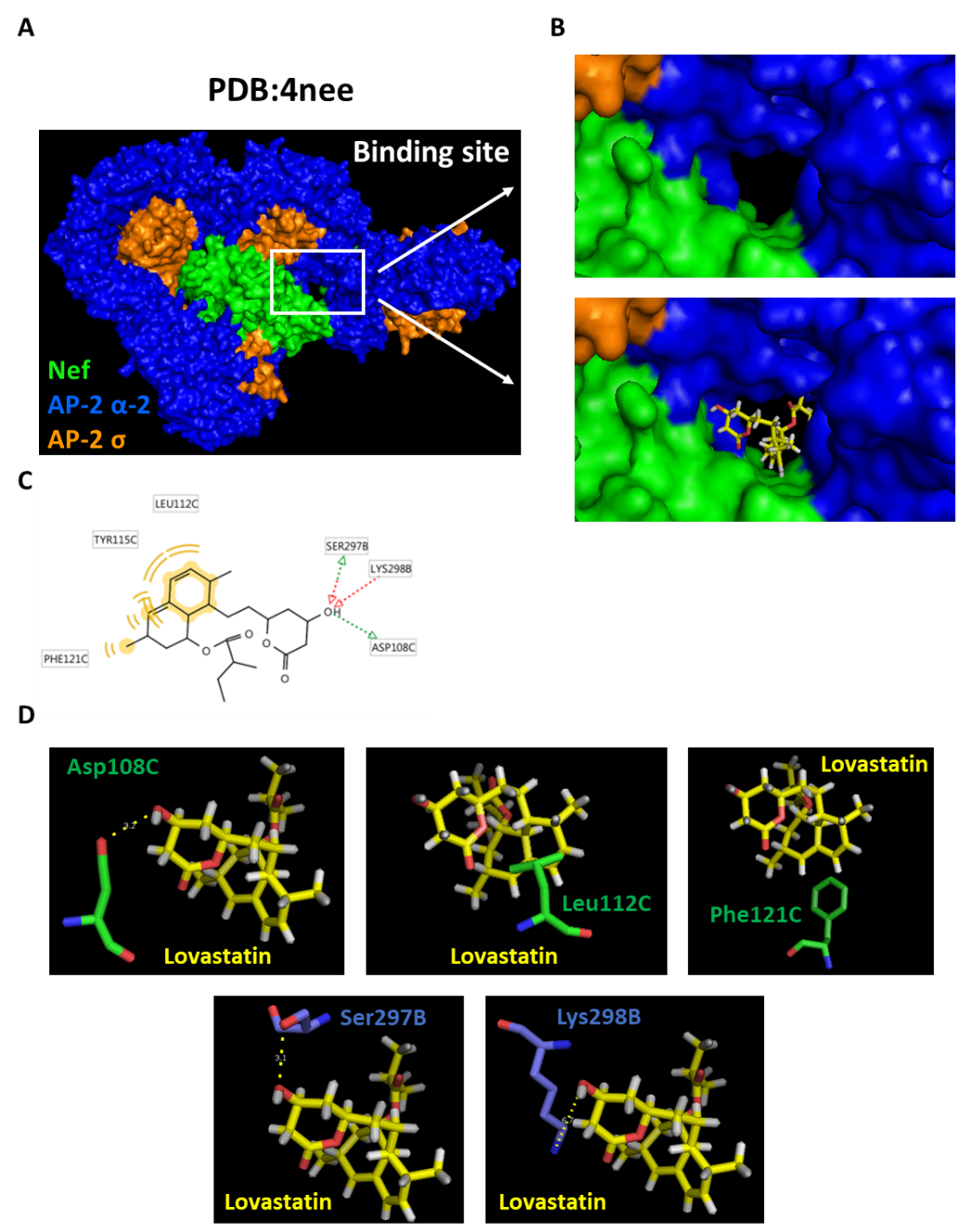
**

**Supplementary Figure 7.** **Molecular docking studies of lovastatin with the crystal structure of HIV-1 Nef in complex with the AP-2 alpha/sigma2 hemicomplex.** The overview of Nef in complex with the AP-2 alpha/sigma2 hemicomplex (PDB: 4NEE): the C, E, H and K chains in green belong to Nef; the A, B, G and J chains in blue belong to AP-2 complex subunit alpha-2; and the D, L, F and I chains in orange belong to AP-2 complex subunit sigma **(A)**. A close-up view and the pose of lovastatin in binding pocket **(B)**. The pharmacophore model of the interaction between lovastatin with Nef–AP-2 complex **(C)**. The specific binding model details: the carbon backbone of lovastatin is in yellow, the Asp108, Leu112, and Phe121 of chain C carbon backbone is in green and the Ser297 and Lys298 of chain B carbon backbones is in light blue **(D)**.

**Supplementary Figure 8**

**
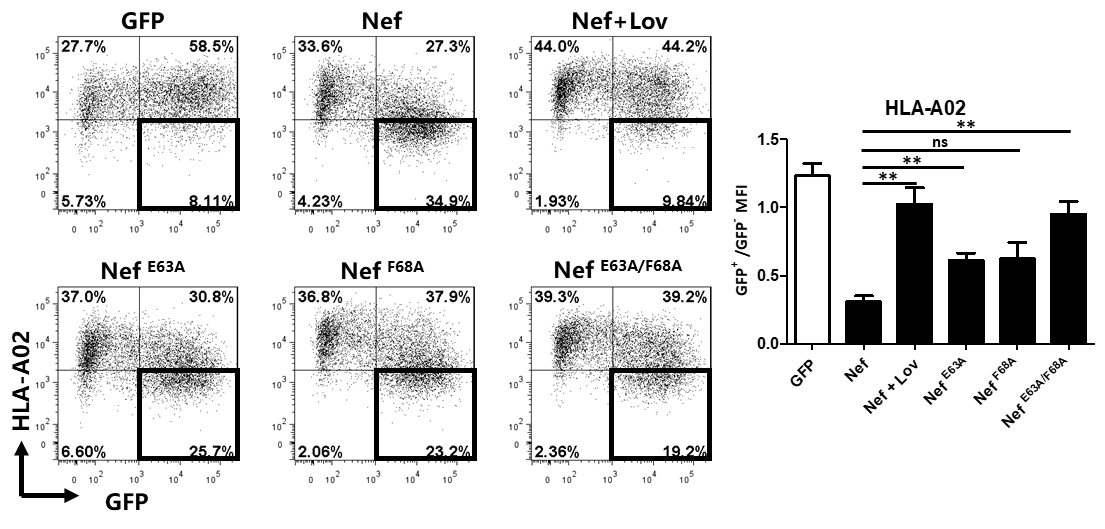
**

**Supplementary Figure 8. The mutations on E63 and F68 attenuate Nef functions on MHC-I downmodulation.** HEK293T cells were transfected with pcDNA3.1-IRES-GFP, pcDNA3.1-Nef-IRES-GFP or Nef mutants (800 ng per well). Twelve hours after transfection, the cultures were treated with lovastatin (4 μM) or vehicle. Forty-eight hours after transfection, the percentages of the GFP^+^ MHC-I ^-^ cells and the ratios of MHC-I MFI on GFP^+^ to GFP^-^ cells were analyzed by flow cytometry. Data show the means ± standard deviations in three independent experiments. P-values were calculated using the two tailed unpaired Student’s t-test with equal variances, n = 3. *p < 0.05, **p < 0.01, ***p < 0.001.

**Supplementary Figure 9**

**
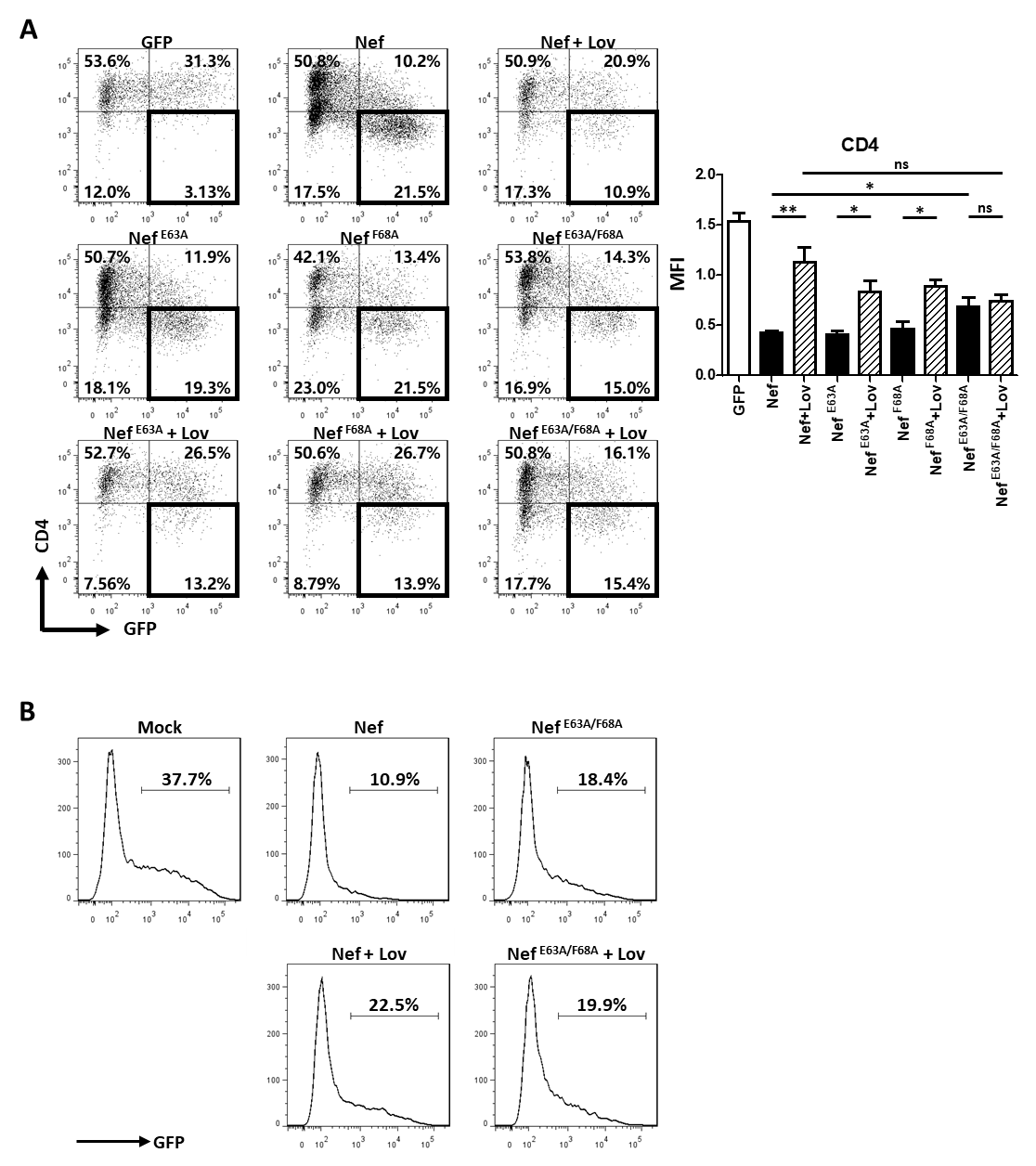
**

**Supplementary Figure 9. The mutations on E63 and F68 attenuate Nef functions on CD4 downmodulation and SERINC5 antagonism.** TZM-bl cells were transfected with pcDNA3.1-IRES-GFP, pcDNA3.1-Nef-IRES-GFP or Nef mutants (800 ng per well). Twelve hours after transfection, the cultures were treated with lovastatin (4 μM) or vehicle. Forty-eight hours after transfection, the percentages of the GFP^+^ CD4 ^-^ cells and the ratios of MHC-I MFI on GFP^+^ to GFP^-^ cells were analyzed by flow cytometry **(A)**. Twelve hours after transfection of SERINC5-GFP/GFP (500 ng per well) and HA-Nef plasmids (500 ng per well) into HEK293T cells, the cultures were treated with lovastatin (4 μM) or vehicle. FACS analysis was performed for the percentages of the GFP ^+^ cells at 48 hours after transfection **(B)**. Data show the means ± standard deviations in three independent experiments. P-values were calculated using the two tailed unpaired Student’s t-test with equal variances, n = 3. *p < 0.05, **p < 0.01, ***p < 0.001.

**Supplementary Figure 10**

**
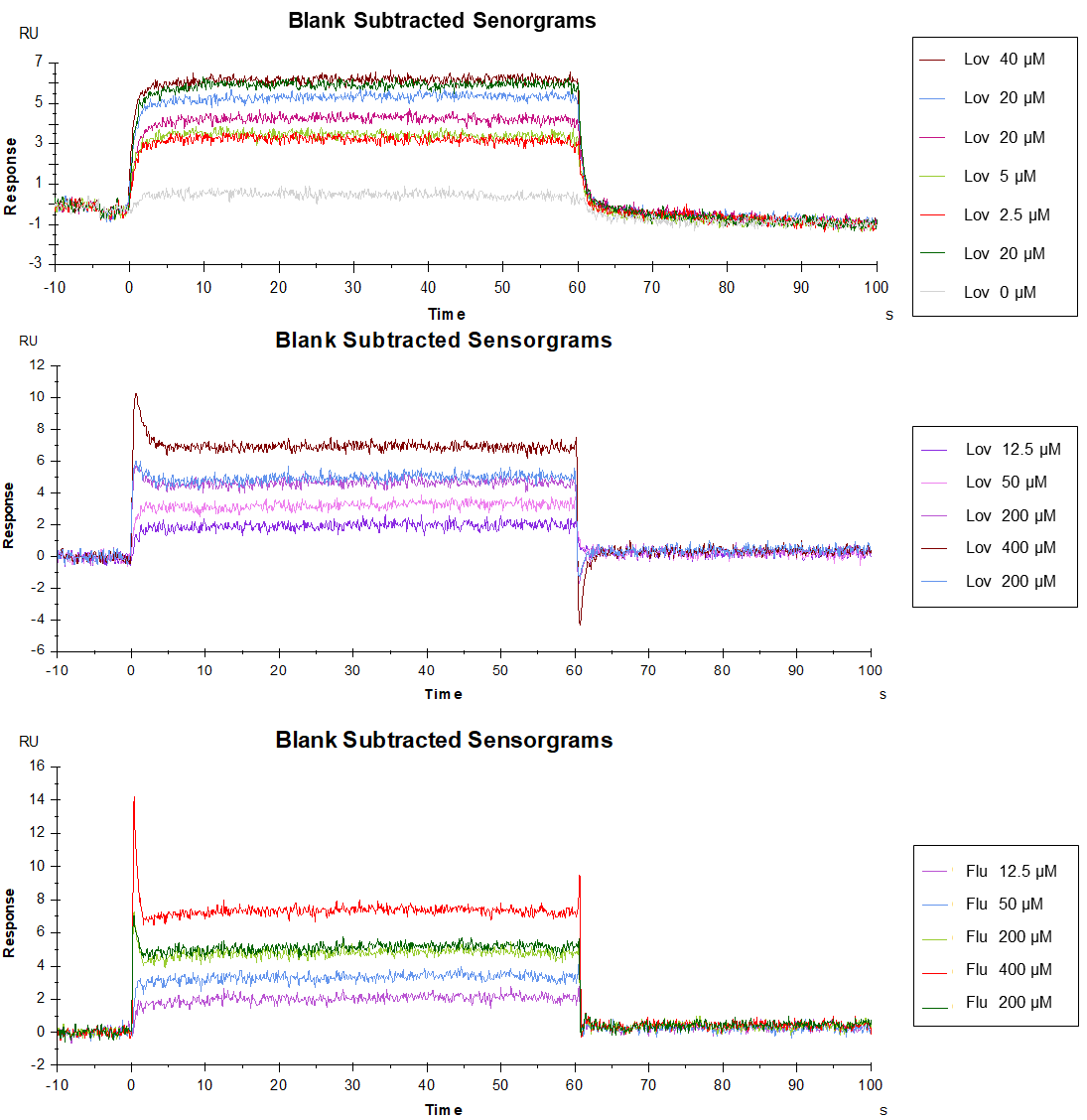
**

**Supplementary Figure 10.** Surface plasmon resonance experiments were performed to measure the binding affinity of lovastatin on Nef or Nef ^E63A/F68A^ and fluvastatin on Nef with a BIAcore T100 Biosensor System.
